# Supplementary material for: Distinct gradients of cortical architecture capture visual representations and behavior across the lifespan
Source: eLife. 2026 Oct 30;13:RP102095. doi: 10.7554/eLife.102095 (PMC13722415; doi:10.7554/eLife.102095)
Supplement: Peer review history

# Editor report: eLife Assessment

**DOI:** 10.7554/eLife.102095.3.sa0

**Authors:** Tessa Dekker

**Affiliations:** University College London United Kingdom

**Keywords:** Compelling, Valuable

This **valuable** study draws on large-scale multimodal MRI measurements of human brain structure across the lifespan to offer a new perspective on visual cortex architecture. The data provide **compelling** evidence for two cortical architectural gradients that show distinct functional, cytoarchitectural, behavioural, and lifespan profiles. One gradient captures a broad early-to-higher-level visual cortical hierarchy in which cortical thickness tissue density covary; the other reflects more localised divergence from this relationship, notably predicting putative anterior temporal visual field representations that have not previously been described.

# Referee report: Reviewer #1 (Public review)

**DOI:** 10.7554/eLife.102095.3.sa1

Summary:

The manuscript uses large-scale existing datasets that span almost the full range of human life (5-100 years) to identify two distinct architectural cortical gradients within visual cortex. These gradients are distinct in that in one cytoarchitecture and myeloarchitecture converge and in the other they diverge. The authors tested whether these gradients mapped onto known functional properties of visual cortex, as well as accounting for visual behaviours that are impacted throughout the lifespan. The manuscript also reports the identification of a hitherto unknown cluster of visual field maps in the anterior temporal lobe.

Strengths:

A major strength of the current manuscript is the use of large-scale measurements of human brain structure throughout the lifespan, courtesy of the Human Connectome Project Initiative. The scope of this cross-sectional analysis would be rare, if not impossible to achieve through an individual project.

The approach employed holds promise for assessing the link between large-scale anatomical gradients in the brain and functional/behavioural properties. The current manuscript focuses on visual cortex, but the approach could easily be implemented across the brain in general.

Weaknesses:

While the evidence for a new topographic visual field map cluster in the anterior temporal lobe is less convincing than for clusters in posterior cortex, new analyses strengthen the claim for a visuospatially tuned cluster that shared signatures of topographically organised clusters (e.g., contralateral representations) but might lack clear evidence, at present, for such topography. Investigation of how age-related and SNR confounds contribute to gradients and their life-span development could be expanded.

Comments on revised version.

The authors have taken the comments onboard and performed a number of analyses that strengthen the argument for these clusters being visuospatial in nature. I appreciate the additional analyses and effort. It may be helpful to discuss the evidence for contralateral biases in the absence of clear topographic maps in cortex in the context of what others have terms visuospatial coding (Groen et al., 2021, TiCS) where just such a mechanism is described.

# Author comment: Author response

**DOI:** 10.7554/eLife.102095.3.sa2

**Authors:** Xiayu Chen, Xingyu Liu, Patricia Maria Hoyos, Edan Daniel Hertz, Jewelia Yao, Zonglei Zhen, Jesse Gomez

**Affiliations:** Beijing Normal University Beijing China; University of Pennsylvania Beijing China; Princeton University Princeton United States; Princeton University Princeton United States; Princeton University Princeton United States; Beijing Normal University Beijing China; Princeton University Princeton United States

The following is the authors’ response to the original reviews.

**Public Reviews:**

**Reviewer #1 (Public review):**

Weaknesses:

While the evidence in favour of the two gradients largely supports the claims, the evidence for a new visual field map cluster in the anterior temporal lobe falls short of the level used historically when identifying visual field maps in the visual cortex and is, at present, not convincing. More specifically, the progressions of polar angle within the putative anterior lobe cluster are highly variable across subjects. Few subjects have convincing polar angle reversals at either the horizontal or vertical meridians. In other cases, a putative border is shown that spans different polar angles, which does not align with the accepted definitions for visual field maps in the cortex.

We agree with the reviewer that more evidence could be provided in support of retinotopic representations within the anterior temporal lobe. We have performed a number of new analyses to further explicate the receptive field properties of this anterior temporal lobe visual representation. We have pasted updated Figure 2e-i. We have added additional participants, increasing the total number from N=12 to N=21. In panel g, we show that in this larger group, we can still observe pRFs that are about 3x larger than those in early visual cortex, and that the relationship between their size and eccentricity shows the expected steeper slope compared to these early representations. In this new participant group, we also illustrate the visual field coverage of the left and right anterior temporal lobe representations (panel h). As expected, the left hemisphere pRFs largely sample the right visual field, and right hemisphere pRFs largely sample left visual space. One can also see that both the upper and lower visual fields are sample quite evenly, consistent with the hemi-field representation of visual field maps observed in earlier visual cortex. To quantify whether there is a left-right contralateral bias in the sampling of visual space (and to test whether such a bias is significantly different in each hemisphere), we calculated for each pRF a laterality index as previously defined by Sheremata and Silver (2015) according to the equation below:

$$\displaystyle \left[\frac{1}{\sqrt{\pi}} \int_{\frac{x_{0}}{\sqrt{2} \sigma}}^{+\infty} e^{-t^{2}} d t\right]$$

Where resulting values of 1 mean the pRF is contralateral, 0.5 is no laterality bias, and 0 is ipsilateral bias. Additionally, we input pRF sigma values that were adjusted for the non-linearity exponent as defined by Kay et al. (2013). For the purposes of visual comparison, we subtracted 0.5 from index values so that resulting laterality scores were relative to 0 to represent the center of the visual field, and then values were inverted with a -1 scalar so that left hemisphere pRF laterality index values are plotted on the right side of space, and the right hemisphere on the left as shown in panel i. The laterality index was calculated for each pRF for a given participant and then averaged within that participant to result in a single mean laterality index for the left hemisphere pRFs and a single index for their right hemisphere pRFs. The histograms illustrated in panel i depict density of participants (kernel smoothed). We find a significant difference between laterality indices with left AT pRFs showing significantly rightward index values compared to right AT pRFs (paired-samples t-test, t(20) = 7.6, p = 2.7 x10^-7^). These data thus offer stronger evidence of a hemifield representation with a contralateral bias, and it should also be noted that there is stronger ipsilateral coverage in these high-level visual pRFs compared to earlier visual field maps like V1, which is consistent visual field maps in latera stages of the visual processing hierarchy as quantified by Mackey et al. (2017).

Lastly, we note that the progression of polar angle values on the cortical surface is certainly not as strikingly topographic as in visual field maps V1 through hV4. This is perhaps a result of the strong ipsilateral visual field coverage in which pRFs whose centers were near or within the ipsilateral field (especially those near the fovea) are not visualized appropriately when using a contralateral colormap. It is also possible that at this very late stage of visually-responsive cortex within entorhinal cortex that retinotopic topography becomes less clear as is the case in higher stages of the dorsal visual stream. To improve visualization, we have created a new Supplemental Figure 6 using a binary color map that colors lower and upper visual field in separate colors and extends into the ipsilateral visual field (pasted below for convenience). We hope that this color map helps to show the upper and lower visual field coverage. While there is a clear radial eccentricity gradient within these AT pRF clusters, and while most participants do show a polar angle gradient that runs perpendicular to this radial eccentricity gradient as expected for a visual field map, we do agree that it is difficult to observe polar angle traversals as clearly as in earlier visual cortex. Nonetheless, the presence of these pRF clusters which show their own distinct eccentricity representation (i.e., a foveal confluence) and a full sampling of the contralateral visual space is still consistent with our anatomical model’s prediction in which PC2 anchor points predict foveal representations shared by visual field map clusters. While the topographic clarity of these representations on the cortical surface is less than earlier visual cortex, the existence of contralateral representations of visual space with a full eccentricity gradient that spans the upper and lower visual field is strongly supported by the data and consistent with our anatomical model’s prediction that there should have been a distinct eccentricity gradient. These findings are also consistent with work showing that the human hippocampus also shows sensitivity to contralateral visual space (Silson et al., 2021) and suggests the hippocampus may inherit this contralateral bias from this entorhinal visual representation. We have updated the manuscript to incorporate these new findings, and refer to these AT clusters as contralateral visual representations, remaining agnostic to whether or not they can be fully defined as topographic maps which can be the focus of future work using smaller voxel sizes to better capture small topographic gradients.

We have revised the manuscript to incorporate these points in the following sections.

Line 466: “We performed pRF mapping on 21 participants with high-contrast, …”

Line 601-625: “To produce maps of visual field coverage (Figure 2h) similar to previous work, … The histograms illustrated in Figure 2i depict density of participants (kernel smoothed).”

Line 236-246: “We find that consistent with its high position within the processing hierarchy, … We find a significant difference in laterality indices between left and right AT pRF’s (pairedsamples t-test, t(20) = 7.6, p = 2.7 × 10-7).”

Line 373-383: “The organization of polar angle in anterior temporal cortex was not as orderly as earlier visual cortex, … in more posterior portions of ventral occipitotemporal cortex.”

**Reviewer #2 (Public review):**

Weaknesses:

(1) The neurobiological model does not take into consideration present knowledge about the microstructural organization of the visual system. This limits the way the results are interpreted correctly. Critical information on the layer-specific myeloarchitecture and cytoarchitecture (and their relation to cortical thickness), as explored for example by Sereno et al. 2013 Cereb Cortex, is missing. There is no information given with respect to how different visual areas differ in their microstructural profile. It is also not mentioned that cortical parcellation is indeed characterized by sharp boundaries between areas, rather than structural gradients, so it remains unclear why focusing on a gradient is of interest. The authors cite the parcellation atlas by Glasser et al. 2016, but do not discuss the rationale of this publication, which was not the definition of gradients, but the definition of sharp boundaries for cortex parcellation. Indeed (as explained below), the results of the authors seem to a large extent to be driven by cortex parcellation, but instead of acknowledging this fact, the authors write (line 179) that "we hypothesize that these local deviations from the canonical thickness and density of cortex underlie the finer-scale division of visual cortex into categorically distinct regions. That is, does the realization of the cortex into distinct regions involve these regions becoming more distinct from a prototypical cortical sheet (i.e., gradient 1)?" - While the first sentence is reasonable, the second sentence is pure speculation ignoring present knowledge on cortical parcellation of this area according to which there is no "prototypical cortical sheet", but each area has its distinct microstructural profile.

We thank the reviewer for this important comment. We first want to point out that we believe there is a conceptual misunderstanding on the part of the reviewer, as we address in our lengthy response below. In this response, we explain that our findings capture what we believe is a novel finding—that variation across participants in the cortical sheet is not random across the spatial expanse of cortex but respects its functional boundaries—which we view as a finding that is complimentary to the current knowledge about the microstructure of visual cortex. It was not our intention to ignore or gloss over this present knowledge, but instead show that variation in these cortical microstructures across brains is not random.

We agree that incorporating current knowledge about the microstructural organization of visual cortex, including its laminar architecture and sharp areal boundaries, is critical for situating our findings within the broader literature. In response, we have added key background information on the relationships among cytoarchitecture, myeloarchitecture, and cortical thickness, as described in previous studies (for example, Maingault et al., 2021; Sereno et al., 2013; Shafee et al., 2015). While our study does not aim to capture layer-specific properties per se, which would require different imaging modalities and higher-resolution data, we focus on spatial properties tangential to the cortical surface.

We first address a concern that the particular parcellation might be driving effects with an analysis showing that we believe our finding is robust to this concern. As suggested by the overall negative covariance observed between cortical thickness and tissue density, we further confirmed this relationship not only across larger visual ROIs, which could potentially reflect effects of arealization, but also within individual ROIs at a finer spatial scale. To avoid potential circularity in ROI definition, we used a visual ROI atlas derived from population-level retinotopy based on independent datasets (Abdollahi et al., 2014). We found that at the global level, cortical thickness and T1w/T2w ratio showed a strong negative correlation across visual ROIs (Fig. 3, revised Supp. Fig. 3a & b). Although only a portion of the visual cortex is clearly delineated in this atlas, we replicated similar results across the entire visual cortex using the MMP atlas (Glasser et al., 2016). At the within-ROI level, we found robust negative correlations between cortical thickness and T1w/T2w ratio across most visual ROIs in both hemispheres, with the notable exception of V1, V2 and VO1, which exhibited a positive relationship, consistent with prior work (for example, Maingault et al., 2021; Sereno et al., 2013; Shafee et al., 2015). These results highlight both common and distinct microstructural profiles across the visual cortex and provide important context for interpreting our data-driven findings.

We also want to address what we think is a conceptual misunderstanding by the reviewer, which likely resulted from a lack of clarity on our part. The reviewer’s confusion likely results from the fact that we theoretically “transposed” the typical PCA analysis such that we get a subject-wise contribution (PC loadings) per participant (also see response to next point), which is how we’re able to relate inter-participant variability in their loadings to behavior in Figure 3. This is also why we refer to a “typical” cortex/cortical sheet because the surface maps being visualized for PC2 can be thought of as a map explaining variance of deviation orthogonal to PC1 (which captures the primary relationship between thickness and T1/T2). Thus, because PC2 is orthogonal to PC1, it captures the spatial pattern in which participants deviate from the primary relationship (e.g., the typical relationship). Therefore, if a given participant is far from the PC1 vector and has high PC2 loading, their cortical sheet is either thicker or more myelinated than predicted by the PC1 relationship and is therefore more distinct from the “typical” or “average” cortical sheet values captured by PC1. We want to emphasize that PCA is agnostic to spatial structure across the cortex. Thus, the fact that deviation from the primary thickness-myelination relationship (i.e. PC2) captured by PC1 had any spatial structure at all is interesting. Furthermore, the fact that the spatial structure of PC2 across the cortical sheet seems to separate visual cortex into its constituent processing streams is also interesting. Therefore, we are not speculating but rather describing the PCA model itself whereby a participant’s loading on PC2 describes their deviation or distinctness from the PC1 relationship. The fact that PC2 has spatial structure on the cortical sheet (which did not have to be true) and the fact that this structure seems to capture broad borders between visual processing streams and field maps is what we find interesting and quantify within the paper. We hope this additional explanation clarifies the broader theoretical thrust of the paper. We view these findings as complimentary to the present knowledge of the microstructural organization of the visual system. Our findings suggest that variability in these microstructural features across participants (PC2) don’t occur randomly across cortex but seem to respect the functional borders of the neural populations of the underlying cortical sheet.

Regarding the concern that our gradient approach may contradict established knowledge of cortical arealization, we would like to clarify that the primary goal of our gradient analysis is not to redefine visual areas, or to go against cortical arealization, but to explore the continuous variation in cortical architecture across brains that may co-exist alongside sharp boundaries which is phenomenon complementary to the arealization. In our study, cortical thickness maps were regressed for curvature before entering any analyses, given the covariance between cortical folding and area borders (Fischl et al., 2008). We acknowledge that cortical parcellation is traditionally characterized by discrete transitions between areas. However, our results suggest that gradients of cortical properties—particularly those shared across participants—may capture supra-areal organizing principles that reflect how distinct regions relate to one another within a broader cortical sheet.

Finally, we agree with the reviewer that the phrase “prototypical cortical sheet” was speculative and potentially misleading. We have removed this language from the manuscript and revised the corresponding discussion.

We have revised the manuscript to incorporate these points in the following sections.

Line 92-94: “Thickness and density maps showed a robust anti-correlation both at the coarse across-area level based on an independent parcellation and at the finer within-area level, except in primary regions (Figure S3a, b).”

Line 350-353: “The convergence pattern, arising from the negative correlation between thickness and density, is consistent with previous findings and may support the balloon model, whereby cortical thinning is associated with tangential stretching due to myelination.”

Line 188-189: “That is, does the arealization of cortex into distinct regions involve these regions becoming more distinct from a typical cortical sheet (i.e., gradient 1)?”

(2) Instead of building on present, detailed knowledge of brain anatomy and in-vivo cortex parcellation of the visual system and its known relation to visual maps, the authors focus on two metrics of cortex architecture (mean T1/T1 over depth and cortical thickness), and conduct a PCA to explore their shared variance. It needs to be clarified if the PCA was conducted correctly. There is no mention of standardizing the variables, which could bias the results. In addition, in a PCA, all possible features are categorized as vector components, and those are scanned through the samples, hence, one such analysis per vertex. But the authors write "in which participants are features and cortical vertices are samples" and "the thickness and tissue density maps were concatenated". This needs clarification. The architecture of the PCA should be visualized better.

We thank the reviewer for pointing out the need to clarify the PCA methodology. In response, we have revised the Methods section to provide a clearer and more accurate description of our approach.

We also would like to point the reviewer’s attention to Figure 1a, in which the PCA was illustrated graphically. The reviewer’s confusion likely results from the fact that we theoretically “transposed” the typical PCA analysis such that we get a subject-wise contributions (PC loadings) per participant, which is how we’re able to relate inter-participant variability in their loadings to behavior in Figure 3. This is also why we refer to a “typical” cortex/cortical sheet because the surface maps being visualized for PC2 can be thought of as a map explaining variance of deviation orthogonal to PC1 (which captures the primary relationship between thickness and T1/T2). Thus, because PC2 is orthogonal to PC1, it captures the spatial pattern in which participants deviate from the primary relationship (e.g., the typical relationship).

We have revised the manuscript in the following sections.

Line 493-502: “For each hemisphere, individual cortical thickness and T1/T2-weighted ratio maps from all HCP-YA participants—each represented as an M × N matrix, … corresponding participant-wise contributions (i.e., PC loading or individual weights) in pairs.”

(3) Because the PCA only contains two features, PC1 is driven by the positive relationship between cortical thickness and mean T1/T2, whereas PC2 is driven by their negative relationship. Because in the early visual cortex, cortical thickness and mean T1/T2 correlate positively, it naturally follows that PC1 relates to pRF size (but mediated by the actual cortex parcellation). However, it is unclear why this insight is interesting. I also do not share the view that "these findings demonstrate that gradient 1 acts as a global gradient enveloping the entire visual cortex (...) while gradient 2 acts as a local gradient specific to individual visual streams". I think this relationship between cortical thickness and T1/T2 ratio does not have much to do with local and global gradients. But if so, stronger arguments as to why this should be the case should be presented. What the authors make of this result (particularly the discussion starting line 366) is not clear to me. I cannot follow the line of argumentation, which in my view is too far away from the data.

We appreciate the reviewer’s thoughtful comments and agree that, in general, cortical thickness and T1w/T2w ratio tend to be negatively correlated, with early visual areas (i.e., V1 and V2) representing a notable exception—an observation we highlight and support with evidence in R2. Given this overall pattern of correlation, it may seem intuitive to interpret PC1 as capturing a convergent relationship across the two metrics, and PC2 as reflecting their divergence. Alternatively, one can think of PC2 as the orthogonal residuals from the linear relationship between thickness and myelin captured by PC1. In this framework, PC2 is not necessarily the inverse correlation, but instead what is left unexplained through a simple linear model. However, it is important to note that PCA is inherently agnostic to spatial structure, as our PCA operates solely on inter-subject variance. As such, the spatial patterns observed in the resulting component maps are not direct or trivial consequences of the input correlations.

Upon examining the spatial properties of the PCA-derived maps (Fig. 1d), we found that PC1 manifests as a large-scale, low-frequency gradient spanning broad portions of the visual cortex, whereas PC2 exhibits a fine-scale, high-frequency pattern confined to subregions of the visual cortex (quantified in Fig. 1f, g). Our initial use of the terms “global” and “local” may have inadvertently implied functional interpretations beyond our intent. We have revised the manuscript to clarify that these descriptors were intended purely to convey differences in spatial scale based on the observed frequency content of the gradients.

Motivated by the reviewer’s comment, we performed additional analyses to explicitly test whether the PCA components reflect consistent (i.e., global) or variable (i.e., local) relationships across visual ROIs. Specifically, we examined whether the direction and magnitude of PC1 and PC2 scores within each ROI align with the global relationships between cortical thickness and tissue density. As shown in the revised Supp. Fig. 3e, we found that in most ROIs, vertices with high PC1 scores consistently exhibit high cortical thickness and low T1w/T2w ratios, while those with low PC1 scores show the opposite pattern. This within-ROI consistency mirrors the largescale cross-ROI correlation structure (see Supp. Fig. 3a), supporting the interpretation of PC1 as reflecting a large-scale, cortex-wide organizational principle. In contrast, PC2 shows more heterogeneous profiles across ROIs, with peaks and troughs that differ in the two metrics. This variability suggests that PC2 captures more localized, region-specific features.

We have incorporated the results of these new analyses into the Results section to strengthen our argument regarding the spatial scale and cross-regional consistency of the PCA-derived gradients:

Line 102-107: “Within-area analyses further confirmed that PC1/2 represent the consistent/deviating components … while PC2 represents the spatial divergence from this commonality.”

**Recommendations for the authors:**

**Reviewing Editor Comments:**

Through collaborative discussions among the reviewers, we first summarised the key recommendations for enhancing the significance and strengthening the evidence of the work - integrating public reviews and recommendations to authors by each reviewer individually. The individual reviewer recommendations can be found below this.

(1) Modelling component 2

The geodesic model for component 2 is interesting but we can recommend ways to improve the evidence and interpretation (see Reviewer 1 comments). As the polar angle reversals are inconsistent and boundaries ambiguous, the OTS maps do not meet the standard of evidence required for showing a new map. The 181 pRF maps available for these HCP data would provide an independent more powerful test of the OTS map cluster. To further strengthen the evidence for the proposed correspondence of foveal confluences and gradient 2, why not define the geodesic model anchoring points based on retinotopic measures, e.g., using HCP pRF data? About the current anchoring points for the geodesic model, what were the criteria - were they objective to avoid circularity?

We appreciate the reviewer’s suggestion to incorporate the HCP 7T retinotopy dataset as an independent test of the proposed geodesic model and its relation to foveal confluences and gradient 2. We agree in principle that such data could provide a valuable validation resource. However, as detailed in the publication accompanying the HCP 7T retinotopy dataset (Benson et al., 2018), the authors recommend a threshold of 9.8% variance explained to distinguish reliable pRF estimates from noise. As illustrated in their Figure 4, this thresholded pRF data shows poor signal coverage in higher-order visual regions, particularly those along the occipitotemporal sulcus (OTS), where gradient 2 effects are most prominent in our data. This lack of reliable pRF signal in these regions limits the utility of the HCP retinotopy data for anchoring the geodesic model or validating the observed spatial gradients.

To address this limitation, we relied on our in-house data collected using high-contrast, naturalistic images designed to robustly activate high-level visual areas. This approach allowed us to define more complete and consistent topographic patterns in the regions of interest. We have thus expanded the size of this in-house dataset to N=21. We also point the editor’s attention to the response to Reviewer 1’s first comment regarding the visual field maps for a more detailed response to this point. For convenience, we have pasted the Figure 2 e-i panels in which we conduct additional analyses showing that these anterior temporal pRF clusters tile contralateral visual space as one might expect (Fig 2h), and significantly differ across hemispheres in their laterality bias (Fig 2i). We have revised the manuscript accordingly.

To mitigate the concern of circularity in defining the geodesic model’s anchor points, we conducted a split-half cross-validation. Anchors were defined on one half of the participants and used to predict the PC2 map in the other half. The PC2 maps across the two halves were highly similar (r = 1.00, p < 0.001), indicating strong reliability. Importantly, the cross-predicted geodesic model accounted for a significant portion of variance (r^²^ = 0.23) in the held-out PC2 map, suggesting that the geodesic organization is not an artifact of overfitting or circular reasoning. We have revised the manuscript accordingly:

Line 139-142: “A split-half cross-validation yielded similar results, … underlying the spatial organization of PC2.”

(2) Speculation about prototypical cortical sheet

You hypothesise that gradient 1 characterises a global "prototypical cortical sheet" characteristic, with gradient 2 reflecting that regions become more distinct from this prototype. There is an alternative simpler possibility: the data can be explained by the stronger relationship between cortical thickness and T1/T2 ratio in early compared to late sensory areas, as can for example be seen in Glasser et al. 2016 Nature, Figure 4. We recommend omitting or balancing the statement about a "prototypical" cortex, and integrating findings on cortex parcellation and the view that sharp boundaries characterize transitions between high and low T1/T2 and cortical thickness areas.

Please see R2 for reviewer #2

(3) Confounds

We'd like to see more data to understand the contributions of data quality to these results. For the component 1 gradient specifically, could its features be influenced by spatial SNR inhomogeneities? Could the developmental effects for both gradients be explained by lower SNR and other data quality markers in younger and older participant data? We missed appropriate tests that gradients develop differently across age, controlling for such confounds (Reviewer 1 comments).

Regarding the reviewer’s concern about the component 1 gradient, we believe it is unlikely to be merely a consequence of uneven spatial SNR. Our findings are consistent with previous histological studies demonstrating systematic variations in cortical architecture—specifically, thinner cortex (Wagstyl et al., 2020) and higher myelin content (Dinse et al., 2015) in occipital compared to ventral visual regions. This correspondence between in vivo MRI-derived measures and postmortem histology suggests that the large-scale organization captured by PC1 is grounded in biologically meaningful cortical architecture, and not an artifact of SNR variability.

To statistically assess whether the two PCs show different developmental trajectories across age, we performed an ANOVA with age, LC, and their interaction as factors on LC’s similarity to PC (i.e., r ~ age + LC + age × LC). Significant age × LC interactions were observed in the developmental (HCPD: F_1,118_ = 257.01, p < .001) and aging (HCPA: F_1,132_ = 263.85, p < .001) cohorts, but not in the young adult cohort (HCPYA: F_1,202_ = 0.02, p = 0.80). These findings indicate that the two gradients show distinct age-related changes during development and aging but remain stable in young adulthood. We have revised the manuscript accordingly:

Line 313-327: “Examining the correlation between the young adult gradient and LC … F_1,132_ = 263.85, p < 0.001.”

(4) Implementation of PCA

The manuscript raises questions about the correct implementation of the PCA - please clarify that the variables were first standardised to enable fair weightings, and visualise the PCA matrix in more detail than in Figure 1a to ensure the samples and features are correctly defined (Reviewer 2).

Please see R3 for reviewer #2

References

Abdollahi, R. O., Kolster, H., Glasser, M. F., Robinson, E. C., Coalson, T. S., Dierker, D., Jenkinson, M., Van Essen, D. C., & Orban, G. A. (2014). Correspondences between retinotopic areas and myelin maps in human visual cortex. NeuroImage, 99, 509–524. https://doi.org/10.1016/j.neuroimage.2014.06.042

Benson, N. C., Jamison, K. W., Arcaro, M. J., Vu, A., Glasser, M. F., Coalson, T. S., Van Essen, D. C., Yacoub, E., Ugurbil, K., Winawer, J., & Kay, K. (2018). The HCP 7T Retinotopy Dataset: Description and pRF Analysis. https://doi.org/10.1101/308247

Dinse, J., Härtwich, N., Waehnert, M. D., Tardif, C. L., Schäfer, A., Geyer, S., Preim, B., Turner, R., & Bazin, P.-L. (2015). A cytoarchitecture-driven myelin model reveals area-specific signatures in human primary and secondary areas using ultra-high resolution in-vivo brain MRI. NeuroImage, 114, 71–87. https://doi.org/10.1016/j.neuroimage.2015.04.023

Fischl, B., Rajendran, N., Busa, E., Augustinack, J., Hinds, O., Yeo, B. T. T., Mohlberg, H., Amunts, K., & Zilles, K. (2008). Cortical Folding Patterns and Predicting Cytoarchitecture. Cerebral Cortex, 18(8), 1973–1980. https://doi.org/10.1093/cercor/bhm225

Glasser, M. F., Coalson, T. S., Robinson, E. C., Hacker, C. D., Harwell, J., Yacoub, E., Ugurbil, K., Andersson, J., Beckmann, C. F., Jenkinson, M., Smith, S. M., & Van Essen, D. C. (2016). A multimodal parcellation of human cerebral cortex. Nature, 536(7615), 171–178. https://doi.org/10.1038/nature18933

Kay, K. N., Winawer, J., Mezer, A., & Wandell, B. A. (2013). Compressive spatial summation in human visual cortex. Journal of Neurophysiology, 110(2), 481–494. https://doi.org/10.1152/jn.00105.2013

Mackey, W. E., Winawer, J., & Curtis, C. E. (2017). Visual field map clusters in human frontoparietal cortex. eLife, 6, e22974. https://doi.org/10.7554/eLife.22974

Maingault, S., Pepe, A., Mazoyer, B., Tzourio-Mazoyer, N., & Crivello, F. (2021). Characterization of late structural maturation with a neuroanatomical marker that considers both cortical thickness and intracortical myelination. https://doi.org/10.1101/2021.02.24.432645

Sereno, M. I., Lutti, A., Weiskopf, N., & Dick, F. (2013). Mapping the Human Cortical Surface by Combining Quantitative T1 with Retinotopy†. Cerebral Cortex, 23(9), 2261–2268. https://doi.org/10.1093/cercor/bhs213

Shafee, R., Buckner, R. L., & Fischl, B. (2015). Gray matter myelination of 1555 human brains using partial volume corrected MRI images. NeuroImage, 105, 473–485. https://doi.org/10.1016/j.neuroimage.2014.10.054

Sheremata, S. L., & Silver, M. A. (2015). Hemisphere-Dependent Attentional Modulation of Human Parietal Visual Field Representations. The Journal of Neuroscience, 35(2), 508–517. https://doi.org/10.1523/JNEUROSCI.2378-14.2015

Silson, E. H., Zeidman, P., Knapen, T., & Baker, C. I. (2021). Representation of Contralateral Visual Space in the Human Hippocampus. The Journal of Neuroscience, 41(11), 2382–2392. https://doi.org/10.1523/JNEUROSCI.1990-20.2020

Wagstyl, K., Larocque, S., Cucurull, G., Lepage, C., Cohen, J. P., Bludau, S., Palomero-Gallagher, N., Lewis, L. B., Funck, T., Spitzer, H., Dickscheid, T., Fletcher, P. C., Romero, A., Zilles, K., Amunts, K., Bengio, Y., & Evans, A. C. (2020). BigBrain 3D atlas of cortical layers: Cortical and laminar thickness gradients diverge in sensory and motor cortices. PLOS Biology, 18(4), e3000678. https://doi.org/10.1371/journal.pbio.3000678
